# Unlike Drosophila elav, the *C. elegans* elav orthologue *exc-7* is not panneuronally expressed

**DOI:** 10.17912/micropub.biology.000189

**Published:** 2019-10-30

**Authors:** Kenneth Pham, Oliver Hobert

**Affiliations:** 1 Columbia University, Department of Biological Sciences, HHMI, New York, NY

**Figure 1:**
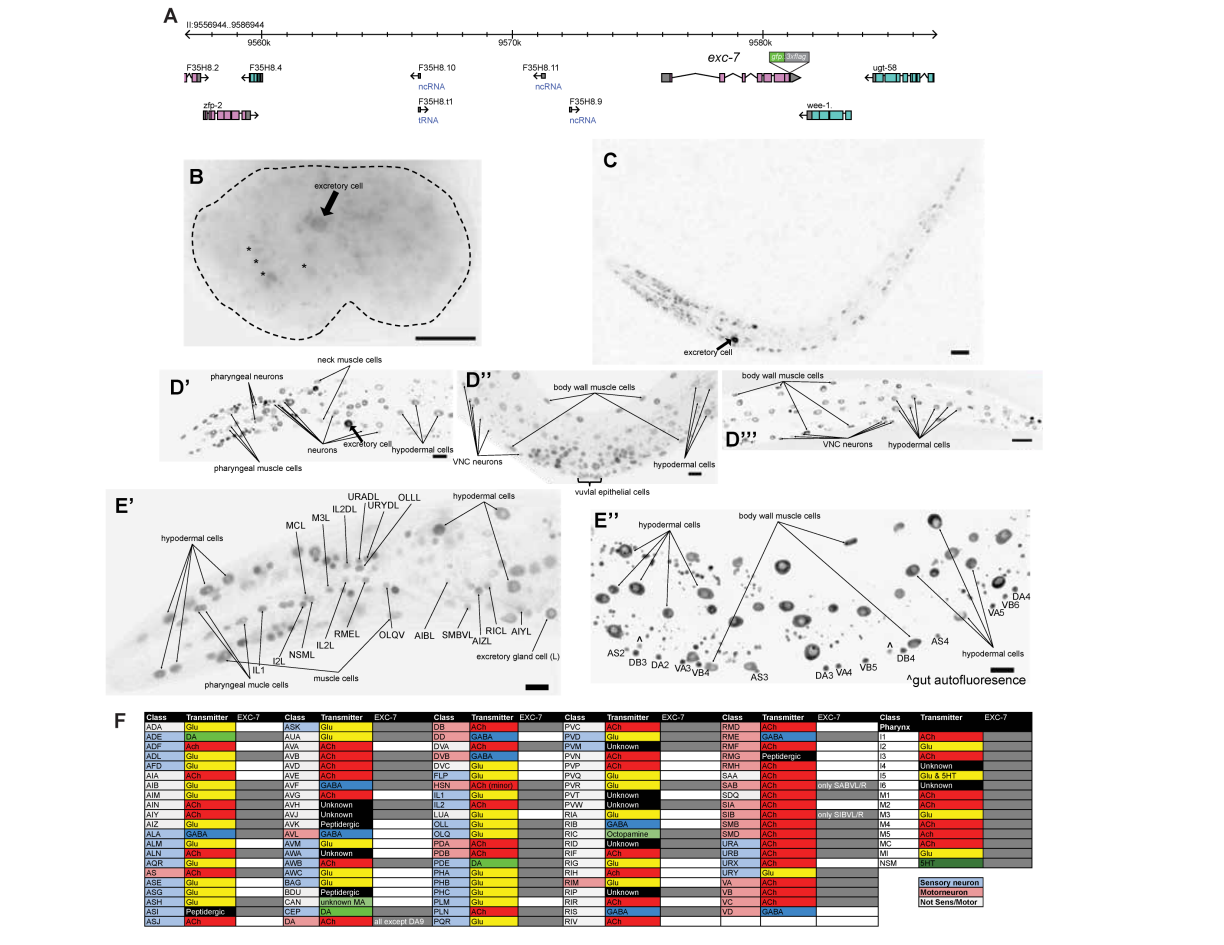
Expression pattern of *exc-7::gfp* in the OH16020 strain. *exc-7* was endogenously tagged at its genomic locus with a *gfp::3xflag* tag (A). Embryonic expression (B) was observed in the excretory canal cell (arrow) and other unidentified cells (*). Broad expression was observed in L1 (C) and young adult head (D’), mid body (D’’), and tail (D’’’), including the excretory canal cell at both stages (arrow). In young adult, individual neurons expressing *exc-7* were identified in the head (E’) and in the ventral nerve cord (E’’). All *exc-7* expressing neurons are listed in panel (F) (grey box). Scale bar, 10 µm.

## Description

We are interested in identifying genes that are expressed in a panneuronal manner throughout the nervous system (Stefanakis *et al.*, 2015). The *Drosophila* *elav* locus is a panneuronally expressed RNA binding protein (Campos *et al.*, 1987; Robinow and White, 1988). Elav protein staining is routinely used in *Drosophila* to identify neurons and cis-regulatory control regions from the *elav* locus are routinely used as panneuronal Gal4 drivers (Berger *et al.*, 2007; Luo *et al.*, 1994; O’Neill *et al.*, 1994; Osterwalder *et al.*, 2001; Robinow and White, 1991). Based on sequence homology, the *C. elegans exc-7* locus is the sole *C. elegans* orthologue of *elav* (Fujita *et al.*, 2003; Fujita *et al.*, 1999; Loria *et al.*, 2003; Samson, 2008). Previous expression pattern analyses have shown that *exc-7* is expressed only in a subset of neurons of the nervous system (the expressing neurons were mostly unidentified) (Fujita *et al.*, 1999; Loria *et al.*, 2003). However, reporter gene constructs previously used to infer *exc-7* expression did not contain all intergenic region of the large *exc-7* locus. Therefore, the possibility remained that through the use of more distal cis-regulatory elements, *C. elegans exc-7* could also be panneuronally expressed, like its fly orthologue. To address this possibility, we tagged endogenous *exc-7* with *gfp::3xflag* at its C-terminus using CRISPR/Cas9 genome engineering (Dokshin *et al.*, 2018) and examined its expression. We cloned *gfp* from the *che-1(ot856[che-1::gfp]*) allele (Leyva-Diaz and Hobert, 2019), inserted it into the pMiniT 2.0 vector (NEB), and used that resulting plasmid for subsequent cloning of the *gfp* tag.

Embryonic expression of *exc-7* was first observed at the bean stage. By reverse lineaging with use of SIMI-Biocell software (Schnabel *et al.*, 1997), we confirm the identity of one of the expressing cells at this stage as the excretory canal cell (Fig. 1B, arrow). In L1 animals, broad expression in the head, ventral nerve cord (VNC), and tail was observed (Fig. 1C). In young adults, expression is notably observed in vulva cells (Fig. 1D’’). In the nervous system specifically, expression is observed in many neurons throughout the body (Fig. 1D’-D’’’), but unlike *Drosophila* Elav, *exc-7::gfp* it is not panneuronally expressed. We used the NeuroPAL transgene (https://www.biorxiv.org/content/10.1101/676312v1) to individually identify each neuron in which *exc-7* is expressed in the young adult worm. Sites of expression are listed in Fig. 1F and some examples of neuronal expression are shown in Fig. 1E’. Expression in all neurons is at least several fold more intense than UPN::NLS::TagRFP-T signal from NeuroPAL. We confirmed previously reported expression in cholinergic VNC MNs, but absence of GABAergic VNC MNs (Fig. 1E’’), consistent with previous reports (Fujita *et al.*, 1999; Loria *et al.*, 2003) and consistent with *exc-7* functioning in cholinergic, but not GABAergic neurons to control alternative splicing (Norris *et al.*, 2014). *exc-7::gfp* is also expressed in some non-neuronal cell types, including muscle and hypodermis, but not in the gut (Fig. 1D’-D’’’). A previous report showed that *exc-7* is only transiently and weakly expressed in the excretory cell, which, based on *exc-7’s* excretory mutant phenotype, has puzzled researchers (Fujita *et al.*, 2003). We find that the *gfp* tagged *exc-7* locus is strongly and continuously expressed in the excretory canal cell (Fig. 1B-D’, arrow). We conclude that unlike its fly orthologue *elav*, *exc-7* is not a panneuronally expressed gene.

## Reagents

OH16020*: exc-7(ot970[exc-7::gfp::3xflag])*

The strain is available through the CGC.

## References

[R1] Berger C, Renner S, Lüer K, Technau GM (2007). The commonly used marker ELAV is transiently expressed in neuroblasts and glial cells in the Drosophila embryonic CNS.. Dev Dyn.

[R2] Campos AR, Rosen DR, Robinow SN, White K (1987). Molecular analysis of the locus elav in Drosophila melanogaster: a gene whose embryonic expression is neural specific.. EMBO J.

[R3] Dokshin GA, Ghanta KS, Piscopo KM, Mello CC (2018). Robust Genome Editing with Short Single-Stranded and Long, Partially Single-Stranded DNA Donors in *Caenorhabditis elegans*.. Genetics.

[R4] Fujita M, Hawkinson D, King KV, Hall DH, Sakamoto H, Buechner M (2003). The role of the ELAV homologue EXC-7 in the development of the Caenorhabditis elegans excretory canals.. Dev Biol.

[R5] Fujita M, Kawano T, Ohta A, Sakamoto H (1999). Neuronal expression of a Caenorhabditis elegans elav-like gene and the effect of its ectopic expression.. Biochem Biophys Res Commun.

[R6] Leyva-Díaz E, Hobert O (2019). Transcription factor autoregulation is required for acquisition and maintenance of neuronal identity.. Development.

[R7] Loria PM, Duke A, Rand JB, Hobert O (2003). Two neuronal, nuclear-localized RNA binding proteins involved in synaptic transmission.. Curr Biol.

[R8] Luo L, Liao YJ, Jan LY, Jan YN (1994). Distinct morphogenetic functions of similar small GTPases: Drosophila Drac1 is involved in axonal outgrowth and myoblast fusion.. Genes Dev.

[R9] Norris AD, Gao S, Norris ML, Ray D, Ramani AK, Fraser AG, Morris Q, Hughes TR, Zhen M, Calarco JA (2014). A pair of RNA-binding proteins controls networks of splicing events contributing to specialization of neural cell types.. Mol Cell.

[R10] O'Neill EM, Rebay I, Tjian R, Rubin GM (1994). The activities of two Ets-related transcription factors required for Drosophila eye development are modulated by the Ras/MAPK pathway.. Cell.

[R11] Osterwalder T, Yoon KS, White BH, Keshishian H (2001). A conditional tissue-specific transgene expression system using inducible GAL4.. Proc Natl Acad Sci U S A.

[R12] Robinow S, White K (1988). The locus elav of Drosophila melanogaster is expressed in neurons at all developmental stages.. Dev Biol.

[R13] Robinow S, White K (1991). Characterization and spatial distribution of the ELAV protein during Drosophila melanogaster development.. J Neurobiol.

[R14] Samson ML (2008). Rapid functional diversification in the structurally conserved ELAV family of neuronal RNA binding proteins.. BMC Genomics.

[R15] Schnabel R, Hutter H, Moerman D, Schnabel H (1997). Assessing normal embryogenesis in Caenorhabditis elegans using a 4D microscope: variability of development and regional specification.. Dev Biol.

[R16] Stefanakis N, Carrera I, Hobert O (2015). Regulatory Logic of Pan-Neuronal Gene Expression in C. elegans.. Neuron.

